# Toward More Accurate Diagnosis in Neurofibromatosis Type 1: A Dual-Level Analysis of Clinical and Molecular Data with Exploratory Genotype–Phenotype Correlations in a Romanian Cohort

**DOI:** 10.3390/genes17070843

**Published:** 2026-07-22

**Authors:** Lăcrămioara Ionela Butnariu, Ecaterina Grigore, Thomas Gabriel Schreiner, Ludmila Darie, Setalia Popa, Ioana Grigore

**Affiliations:** 1 Grigore T. Popa University of Medicine and Pharmacy, 700115 Iasi, Romania; thomas.schreiner@umfiasi.ro (T.G.S.); setalia.popa@umfiasi.ro (S.P.); 2 Regional Center for Medical Genetics Iasi, Saint Mary’s Emergency Children Hospital, 700309 Iasi, Romania; 3 Neurology, Saint Mary’s Emergency Children Hospital, 700309 Iasi, Romania; darie.ludmila@gmail.com (L.D.); ioanag74@yahoo.com (I.G.)

**Keywords:** neurofibromatosis type 1, *NF1* gene, genotype–phenotype correlation, novel variant, Romanian cohort, next-generation sequencing, genetic counseling

## Abstract

Background/Objectives: Neurofibromatosis type 1 (NF1) is an autosomal dominant disorder caused by pathogenic variants in the *NF1* gene, characterized by high phenotypic variability. Methods: We present clinical and molecular data from a Romanian cohort of 54 patients initially diagnosed clinically. Results: Phenotypic evaluation (*n* = 54) revealed a high prevalence of café-au-lait macules (100%), Lisch nodules (64.8%), axillary/inguinal freckling (61.1%), and cutaneous neurofibromas (42.6%). Due to financial constraints (genetic testing not covered by the national health system), molecular confirmation by next-generation sequencing (NGS) was possible in only 12 patients (mostly sporadic cases and young children). Genetic testing identified a diverse spectrum of variants, including frameshift (41.7%, *n* = 5), nonsense (33.3%, *n* = 4), missense (16.7%, *n* = 2), and one splicing deletion (8.3%, *n* = 1). A novel complex *NF1* frameshift variant, c.7504_7508delinsC (p.Ser2502Argfs*24) in exon 54, was identified in a patient exhibiting an aggressive phenotype characterized by plexiform neurofibromas, a malignant peripheral nerve sheath tumor (MPNST), and severe skeletal abnormalities. Additionally, a recurrent nonsense variant, *NF1* c.910C>T (p.Arg304*), was detected in two unrelated individuals. Conclusions: The high proportion of sporadic cases (58.3%) in the molecularly tested subgroup underscores the critical role of early genetic screening. By integrating clinical data from 54 patients with the first molecular characterization of NF1 in Romania, this study expands the mutational spectrum and provides preliminary, descriptive insights into genotype–phenotype correlations. It also proposes a cost-effective diagnostic algorithm adapted for resource limited settings and lays the groundwork for future multicenter initiatives. Given the exploratory nature of the molecular subgroup (*n* = 12), all genotype–phenotype observations require validation in larger independent cohorts.

## 1. Introduction

As one of the most common autosomal dominant disorders affecting the nervous system, neurofibromatosis type 1 (NF1) results from pathogenic variants of the *NF1* gene (OMIM 613113) [1] located on chromosome 17q11.2, which provides the instructions for producing the protein neurofibromin. This protein serves as a tumor suppressor by negatively regulating the RAS/MAPK signaling pathway, thereby controlling cell growth and differentiation [1,2].

The refinement of neurofibromatosis type 1 (NF1) and type 2 (NF2) diagnosis has shifted from traditional clinical observation to an integrated approach that combines molecular data with genotype–phenotype correlations [3,4].

**Figure 1:**
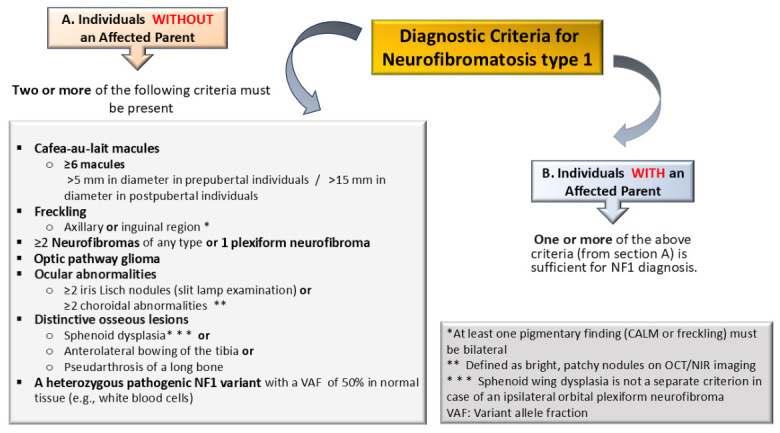
Diagnostic algorithm for neurofibromatosis type 1 based on 2021 revised NIH criteria [3,4,5].

The 2021 revision of the National Institutes of Health (NIH) diagnostic criteria marked a pivotal shift in NF1 management by formally incorporating genetic testing (Figure 1). This integration facilitates earlier and more accurate diagnosis, enables reliable differentiation from phenotypically similar conditions such as Legius syndrome and mosaic NF1 and refines prognostic accuracy through established genotype–phenotype correlations [5]. Recent studies have further validated the clinical utility of this approach, demonstrating that molecular confirmation reduces diagnostic uncertainty and guides personalized patient management [6].

The diagnostic landscape of NF1 has been transformed by the advent of next-generation sequencing (NGS) and multiple ligation-dependent probe amplification (MLPA), which together enable the detection of pathogenic variants in over 95% of cases [7,8]. This high detection rate is particularly critical for identifying segmental or mosaic forms of NF1, which often present with localized pigmentary findings, such as café-au-lait macules, and may otherwise evade clinical diagnosis [9]. For cases where initial DNA sequencing from blood remains inconclusive, complementary approaches such as RNA studies and MLPA are recommended to identify deep intronic mutations that affect splicing or large genomic deletions that are not captured by standard sequencing techniques [8].

Historically, NF1 has been characterized by exceptionally high inter- and intrafamilial phenotypic variability, which led to the prevailing view that the genotype could not predict the clinical course. However, the aggregation of large patient cohorts and advanced molecular analysis has begun to challenge this paradigm, revealing specific and statistically significant genotype–phenotype correlations [9,10,11].

These emerging correlations transcend the traditional view that phenotypes are unrelated to the genotype and are beginning to inform prognostic counseling and clinical management [12].

Several well-established correlations have now been described. Large deletions encompassing the entire NF1 gene (accounting for approximately 5–10% of all cases) are associated with a particularly severe, early-onset phenotype. Affected individuals typically present with a higher overall tumor burden, characteristic facial features, and significant cognitive impairment [13].

Missense mutations affecting codon 1809 (p.Arg1809) are associated with a consistently milder form of NF1, often lacking the typical cutaneous and plexiform neurofibromas [11]. In contrast, missense variants clustered in the cysteine–serine-rich domain (CSRD), particularly within codons 844–848, are correlated with a more severe phenotype, including a higher prevalence of plexiform and spinal neurofibromas, as well as an increased frequency of cognitive impairments [12].

Beyond the well-characterized 844–848 region, two additional missense hotspots—p.Arg1276 and p.Lys1423 (both in exon 28)—have emerged as significant contributors to severe NF1 presentations [12,14]. Together with p.Met1149 (exon 26), these three nontruncating variants account for approximately 1.8% of unrelated NF1 individuals, as documented in a comprehensive study of 281 patients by Koczkowska and colleagues [14].

Patients carrying p.Arg1276 substitutions exhibit a particularly aggressive clinical course. These variants are associated with a significantly increased frequency of plexiform neurofibromas and symptomatic spinal neurofibromas, alongside a notably high prevalence of cardiovascular abnormalities, particularly pulmonic stenosis [10,15]. Skeletal manifestations, including various bone anomalies, and Noonan-like features are also markedly overrepresented in this population [10,15]. Interestingly, despite this severe multisystem involvement, cutaneous neurofibromas occur less frequently in this group—a paradoxical finding that points to a distinct underlying biological mechanism [15]. A comparably severe clinical picture emerges for p.Lys1423 variants [10,15]. Affected individuals demonstrate significantly elevated frequencies of plexiform and symptomatic spinal neurofibromas, alongside increased rates of bone anomalies, scoliosis, and cardiovascular abnormalities [10,15]. Noonan-like features are also prominent in this group, while Lisch nodules appear less frequently and cognitive difficulties show a trend toward increased prevalence [15].

The recurrent association of both p.Arg1276 and p.Lys1423 with cardiovascular manifestations, especially pulmonic stenosis, underscores the need for targeted clinical surveillance in these populations [10,15]. Concurrently, the diminished frequency of certain classic NF1 stigmata within these subgroups reinforces the imperative for molecularly guided diagnostic strategies that extend beyond conventional clinical criteria [10,15].

A more recent and distinct correlation has been identified involving specific missense variants at codon 1204 (p.Arg1204), which appear to be associated with a notable absence of neurofibromas despite meeting other diagnostic criteria [15].

Integrating these molecular findings into routine clinical practice carries profound implications for patient management [16]. The identification of high-risk mutations, such as those in the 844–848 region, can prompt more intensive surveillance protocols, particularly for malignant peripheral nerve sheath tumors (MPNSTs), which represent a leading cause of mortality in NF1 [12]. Furthermore, the treatment landscape for NF1 has been revolutionized by the advent of targeted therapies. The approval of MEK inhibitors (e.g., selumetinib) for the treatment of symptomatic, inoperable plexiform neurofibromas underscores the critical need for precise molecular characterization to determine patient eligibility and predict therapeutic response [16]. Looking forward, the emergence of novel gene-based therapies aimed at restoring neurofibromin function will likely depend even more heavily on understanding the specific nature of the underlying genetic variant. Despite these significant advances, it is crucial to acknowledge that a substantial portion of the phenotypic variability in NF1 remains unexplained by the constitutional genotype alone [17]. This suggests that the final clinical presentation is likely shaped by a complex interplay of additional factors. These include the specific somatic “second-hit” mechanism that inactivates the remaining wild-type allele in a given cell, the influence of epigenetic modifications, the presence of modifying genetic loci elsewhere in the genome, and stochastic environmental factors [17]. Future research efforts are therefore focused on conducting larger, multicenter, prospective longitudinal studies. Such studies are essential not only to validate and refine the existing genotype–phenotype correlations but also to unravel the contribution of these modifying factors, ultimately paving the way for truly personalized approaches to surveillance and treatment for individuals with NF1 [17].

Although genetic testing for NF1 is already integrated into international diagnostic guidelines, current knowledge regarding genotype–phenotype correlations is primarily based on common recurrent mutations. Private or novel variants remain largely uncharacterized from a clinical perspective [18]. Moreover, most large-scale studies originate from specific populations, and the applicability of their findings is limited by potential ethnic or regional differences in mutational spectrum and disease expressivity [19,20].

Despite these global advances in understanding the molecular landscape of NF1, data regarding East European populations, particularly Romanian cohorts, remain extremely scarce. In countries with developing healthcare infrastructure, universal access to high-throughput genetic testing is often restricted by financial constraints. Consequently, there is an urgent need to establish clinical–molecular workflows that maximize diagnostic yield through targeted genetic testing.

To address this gap, the current study presents the clinical characterization of a cohort of 54 Romanian patients diagnosed with NF1, serving as a comprehensive clinical baseline, and outlines a targeted molecular stratification strategy applied to a subgroup of 12 selected patients with complex, sporadic, or early-onset manifestations. Our primary objectives are to describe the population-specific mutational spectrum and to establish preliminary, exploratory genotype–phenotype correlations based on descriptive case-level observations.

By documenting the first molecular data and offering preliminary, descriptive insights into genotype–phenotype relationships for NF1 patients in Romania, this paper aims to demonstrate how strategic integration of genetic testing may contribute to refining diagnostic accuracy, resolving clinical ambiguity, and supporting personalized management. Furthermore, these exploratory findings may contribute to improving the accuracy of genetic counseling in the local context and may inform risk stratification for severe complications, such as optic pathway gliomas (OPGs) and malignant peripheral nerve sheath tumors (MPNSTs). However, given the exploratory nature of the molecular subgroup (*n* = 12), these observations should be interpreted with caution and require validation in larger independent cohorts.

## 2. Materials and Methods

### 2.1. Study Design and Patient Selection

This study was conducted at two complementary levels. First, we performed a clinical characterization of all patients who met the revised 2021 NIH diagnostic criteria for neurofibromatosis type 1 (NF1) during the study period (2016–2026) at the Department of Neurology and the Regional Center for Medical Genetics, Saint Mary’s Emergency Children Hospital, Iai, Romania.

Second, within a molecularly confirmed subgroup of 12 patients, we report three novel *NF1* variants and provide preliminary, descriptive genotype–phenotype observations. Given the financial constraints in Romania, where NGS is not reimbursed and MLPA funding was discontinued, the molecular subgroup reflects real-world challenges in resource-limited healthcare settings, and our findings should be interpreted as exploratory and hypothesis-generating, requiring validation in larger independent cohorts.

The inclusion criteria for the molecularly confirmed subgroup were strict: (1) detection of a pathogenic or likely pathogenic *NF1* variant by next-generation sequencing (NGS) and/or multiplex ligation-dependent probe amplification (MLPA); (2) absence of an alternative molecular diagnosis (e.g., *SPRED1*, *PTEN*, or *PTPN11* variants); and (3) complete clinical records.

Of the 54 patients who met the clinical criteria, 38 underwent some form of molecular testing. Genetic testing for *NF1* is not fully reimbursed by the Romanian national health system; therefore, NGS was performed only in 11 patients, at the families’ own expense, and all 11 tested positive for a pathogenic or likely pathogenic *NF1* variant. MLPA was initially available free of charge through a national program and was performed in 27 patients; however, only one patient (P10) had a positive result (heterozygous deletion of exon 19). Consequently, 12 patients (11 confirmed by NGS and one by MLPA alone) met the inclusion criteria for the molecularly confirmed subgroup. The remaining 42 patients were excluded: 16 had no genetic testing, and 26 had negative MLPA results without NGS confirmation.

### 2.2. Molecular Genetic Analysis

For 11 of the 12 patients, genetic testing was performed through a targeted gene panel at Blueprint Genetics (Espoo, Finland) using next-generation sequencing (NGS) with Illumina technology (San Diego, CA, USA).

Total genomic DNA was extracted from biological samples using a bead-based method, and DNA quantity was assessed fluorometrically. Qualified genomic DNA samples were randomly fragmented using non-contact, isothermal sonochemistry, followed by ligation of sequencing adapters to both ends of the DNA fragments to prepare a sequencing library. Size selection was performed using a bead based method to ensure optimal template size, and the library was amplified by polymerase chain reaction (PCR). Regions of interest (exons and intronic targets) were captured using a hybridization based target capture method, and library quality was assessed to ensure correct template size and quantity. Ready libraries were sequenced using Illumina’s sequencing by synthesis method with paired end reads (2 × 150 bases). Primary data analysis was performed using Illumina’s proprietary software v2.20 (bcl2fastq), and raw sequencing data were converted to FASTQ format. Sequence reads were mapped to the human reference genome (GRCh37/hg19) using the Burrows-Wheeler Aligner (BWA MEM), and variant annotation was performed using VcfAnno and VEP, with reference to public variant databases including gnomAD, ClinVar, and HGMD. Copy number variations (CNVs), defined as single-exon or larger deletions or duplications, were detected using a proprietary bioinformatics pipeline. For missense variants, in silico prediction tools such as SIFT [21], PolyPhen [22], and MutationTaster [23], were used to assist with variant classification. The pathogenicity potential of identified variants was assessed based on predicted consequence; biochemical properties of the amino acid change; evolutionary conservation; and population frequency data from databases such as the 1000 Genomes Project, gnomAD, ClinVar, and HGMD Professional. The specific ACMG/AMP criteria applied for each variant are detailed in Supplementary Table S1. For one patient (P10), molecular confirmation was performed exclusively using Multiplex Ligation-dependent Probe Amplification (MLPA) at the Molecular Genetics Laboratory in Craiova, Romania, using the SALSA MLPA probemix P081/P082 (MRC Holland, Amsterdam, The Netherlands) and analyzed on a CEQ™ 8000 Genetic Analysis System (AdvaMed, Beckman Coulter, Brea, CA, USA). The MLPA methodology involved denaturation of genomic DNA, hybridization of probes to complementary target sequences, ligation of hybridized probes, and simultaneous amplification using a single pair of PCR primers, with fluorescently labeled products separated and quantified via capillary electrophoresis. Relative peak heights were compared to those of a healthy control to determine exon copy number, identifying a heterozygous deletion of exon 19 in the *NF1* gene. The pathogenicity of the identified variants was assessed according to the American College of Medical Genetics and Genomics and the Association for Molecular Pathology (ACMG/AMP) guidelines [24], with variant interpretation further supported by data from HGMD Professional [25] and ClinVar databases [26].

### 2.3. Clinical Data Collection and Genotype–Phenotype Correlation

Clinical data were collected retrospectively from medical records. For each patient, we documented demographic information, clinical manifestations according to the 2021 revised NIH criteria; family history; and results of paraclinical investigations, including brain MRI, ophthalmological examination, and other relevant imaging studies. Genetic counseling was provided to all families, and family trees were constructed for each patient.

Correlations were established between the identified genetic variants and the corresponding clinical phenotypes. The results were compared with data from the specialized literature, highlighting the particularities of each patient within the context of their family.

### 2.4. Ethical Approval

This study was conducted in accordance with the principles of the Declaration of Helsinki and was approved by the Ethics Committee of Saint Mary’s Emergency Children Hospital, Iași, Romania (Certificate no. 11913/08.04.2026). Written informed consent was obtained from all subjects involved in this study (or from their parents/legal guardians for minors).

## 3. Results

**Figure 2:**
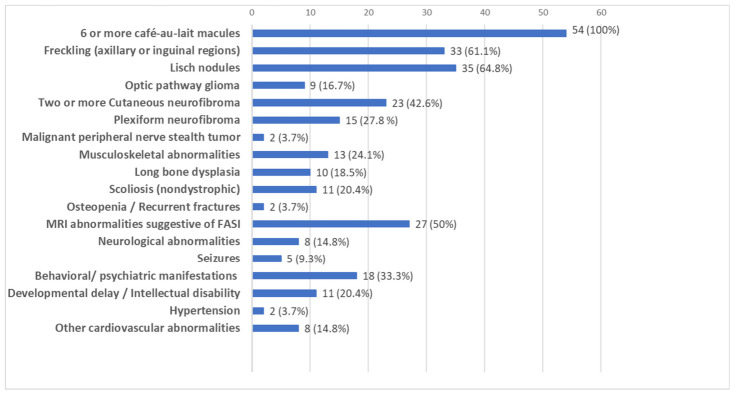
Clinical manifestations in the clinically diagnosed NF1 cohort (*n* = 54).

The initial clinically diagnosed cohort consisted of 54 patients (25 female and 29 male), with ages at diagnosis ranging from 4 months to 17 years (mean age: 9.1 years). All 54 patients (100%) presented with six or more café-au-lait macules. Axillary or inguinal freckling was observed in 33 patients (61.1%), Lisch nodules in 35 patients (64.8%), and cutaneous or subcutaneous neurofibromas in 23 patients (42.6%). Plexiform neurofibromas were present in 15 patients (27.8%), and malignant peripheral nerve sheath tumors (MPNST) were diagnosed in 2 patients (3.7%). Optic pathway gliomas (OPGs) were identified in 9 patients (16.7%). Musculoskeletal abnormalities were reported in 13 patients (24.1%), including long bone dysplasia in 10 patients (18.5%), nondystrophic scoliosis in 11 patients (20.4%), and osteopenia or recurrent fractures in 2 patients (3.7%). Regarding neurological and neuroimaging findings, MRI abnormalities suggestive of focal abnormal signal intensities (FASIs) were present in 27 patients (50.0%). Neurological abnormalities were documented in 8 patients (14.8%), seizures in 5 patients (9.3%), behavioral or psychiatric manifestations in 18 patients (33.3%), and developmental delay or intellectual disability in 11 patients (20.4%). Cardiovascular manifestations included hypertension in 2 patients (3.7%) and other cardiovascular abnormalities in 8 patients (14.8%). The clinical characteristics of these 54 patients are summarized in Figure 2.

**Figure 3:**
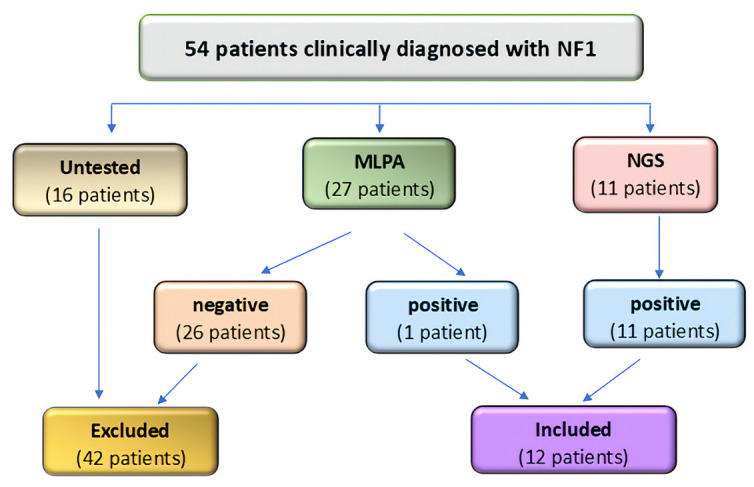
Selection flowchart for the molecularly confirmed NF1 subgroup (*n* = 12). From 54 clinically diagnosed patients, 12 had confirmed pathogenic/likely pathogenic variants (11 by NGS, and one by MLPA). Exclusion reasons: no testing (*n* = 16), negative MLPA without NGS (*n* = 26).

Of the 54 clinically diagnosed patients, 12 met the strict inclusion criteria: complete molecular confirmation by NGS and/or MLPA, absence of alternative molecular diagnoses, and complete clinical records. This subset was selected for detailed genotype–phenotype correlation analysis. Figure 3 illustrates the selection process.

As shown, the final molecularly confirmed cohort comprised 12 patients: 11 with pathogenic or likely pathogenic variants identified by NGS (all subsequently confirmed by MLPA where applicable) and one patient (P10) confirmed by MLPA alone. The remaining 42 patients were excluded due to lack of genetic testing (*n* = 16), negative MLPA without NGS confirmation (*n* = 26), or incomplete clinical records (see the Materials and Methods for details).

The molecularly confirmed cohort consisted of five male and seven female patients, with ages at diagnosis ranging from four months to 13 years (mean age: 5.8 years). All 12 patients carried heterozygous pathogenic or likely pathogenic variants in the *NF1* gene, as detailed in Table 1.

The most frequent variant type was frameshift mutations, accounting for five cases (41.7%) (P01, P04, P06, P09, and P11), followed by nonsense mutations in four cases (33.3%) (P03, P05, P07, and P12), missense mutations in two cases (16.7%) (P02 and P08), and a splicing deletion in one case (8.3%) (P10) (Table 1).

The majority of variants (9 out of 12, 75%) had been previously reported in the literature or databases, while three patients (P04, P06, and P11) carried novel mutations, all of which were frameshift variants. These included a complex frameshift mutation, *NF1* c.7504_7508delinsC (p.Ser2502Argfs*24), in patient P04; the *NF1* c.4967_4968insAGACT (p.Tyr1657Aspfs*22) variant in patient P06; and the *NF1* c.4625del (p.Asn1542Thrfs*11) variant in patient P11, thereby expanding the mutational spectrum of NF1 (Table 1).

A recurrent nonsense variant, *NF1* c.910C>T (p.Arg304*), was detected in two unrelated patients (P03 and P07), suggesting a mutational hotspot in the Romanian population, given its location within a CpG dinucleotide in exon 9 (Table 1).

A positive family history of NF1 was reported in five out of 12 patients (41.7%), while the remaining seven cases (58.3%) were sporadic, indicating a high proportion of de novo mutations. Among sporadic cases, all variant types were represented, including the novel frameshift variants identified in patients P04, P06, and P11 (Table 1).

All 12 patients met clinical diagnostic criteria for NF1, each presenting with six or more café-au-lait macules, a finding that was the most consistent clinical feature across the cohort (Table 2).

Pigmentary manifestations included axillary or inguinal freckling in seven patients (58.3%), while Lisch nodules were identified in four patients (33.3%), all aged eight years or older, consistent with the age-dependent penetrance of this criterion. Regarding tumoral manifestations, cutaneous or subcutaneous neurofibromas were present in three patients (25%), and plexiform neurofibromas were identified in two patients (16.7%). One patient (P04, 8.3%) developed a malignant peripheral nerve sheath tumor (MPNST), representing the most severe tumoral complication in this cohort. Optic pathway glioma (OPG) was diagnosed in two patients (16.7%). Musculoskeletal abnormalities were observed in half of the patients (6 out of 12, 50.0%), including long bone dysplasia, vertebral dysplasia, and dystrophic scoliosis in patient P04, nondystrophic scoliosis in P11, as well as unspecified musculoskeletal abnormalities in P03, P06, P07, and P09. Regarding neurological and neuroimaging findings, MRI abnormalities suggestive of focal abnormal signal intensities (FASIs) were present in nine out of 11 patients with available imaging (81.8%) and were absent only in P06 and P11 (P01 had no available brain MRI).

Unspecified neurological abnormalities were reported in three patients (25.0%), namely P04, P08, and P10, with no seizures recorded in any patient. Behavioral or psychiatric manifestations were present in five patients (41.7%), while developmental delay or intellectual disability was observed in two patients (P06 and P07, 16.7%). Cardiovascular manifestations included hypertension in one patient (P05, 8.3%) and other cardiovascular abnormalities in two patients (P05 and P08, 16.7%) (Table 2).

## 4. Discussion

This study provides a dual-level analysis of NF1 in a Romanian cohort. First, we describe the clinical characteristics of 54 patients meeting the revised 2021 NIH diagnostic criteria, establishing the first clinical baseline for NF1 in Romania. Second, within a molecularly confirmed subgroup of 12 patients, we report three novel *NF1* variants and provide preliminary, descriptive genotype–phenotype observations.

Given the financial constraints in Romania, where NGS is not reimbursed and MLPA funding was discontinued, the molecular subgroup reflects real-world challenges in resource-limited healthcare settings, and our findings should be interpreted as exploratory and hypothesis-generating, requiring validation in larger independent cohorts.

Of the 54 clinically diagnosed patients, 38 underwent some form of molecular testing (27 by MLPA only, 11 by NGS). However, due to the strict inclusion criteria—full molecular confirmation (NGS and/or MLPA), the absence of alternative molecular diagnoses and complete clinical records—only 12 patients met the requirements for definitive genotype–phenotype analysis. Of these, 11 were confirmed by NGS, and one patient (P10) was confirmed by MLPA only. Thus, molecular confirmation was obtained in all 12 cases, reinforcing the reliability of targeted sequencing of *NF1* as a diagnostic tool in individuals meeting the clinical criteria. This finding underlines the importance of molecular testing, especially in cases where the clinical picture is unequivocal, and supports its integration into standard diagnostic pathways—a goal that remains challenging in countries without universal genetic testing coverage.

According to the American College of Medical Genetics and Genomics (ACMG) guidelines [24], nine of the identified variants were classified as pathogenic (P01, P02, P03, P04, P05, P07, P09, and P12), while three variants were designated as likely pathogenic (P06, P08, and P11). In our cohort, all variants were located within the coding sequence of the *NF1* gene, with the majority affecting exons 2, 5, 9, 12, 27, 33, 34, 36, 50, and 54 (Table 1). Although large-scale studies have reported a relatively uniform distribution of *NF1* mutations across the gene without significant clustering in specific exons [27,28], the observed predominance in our series reflects the specific mutational profile of the population analyzed.

**Figure 4:**
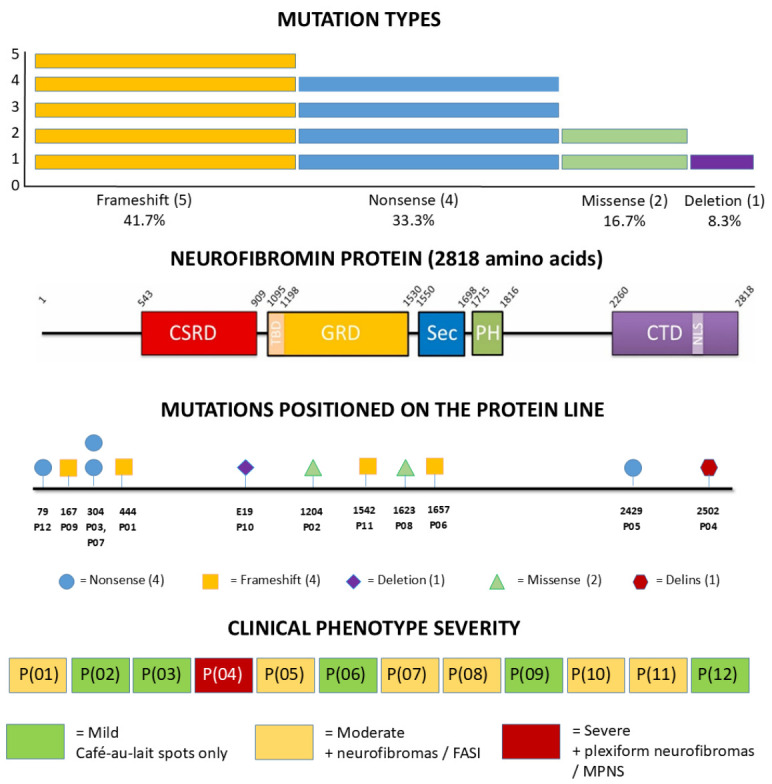
Genotype–phenotype correlations in the molecularly confirmed NF1 subgroup (*n* = 12). The figure integrates mutation type distribution, protein domain localization, and clinical severity. Upper panel: Distribution of mutation types among the 12 patients. Frameshift mutations (yellow) accounted for 5 cases (41.7%), nonsense mutations (blue) for 4 cases (33.3.%), missense (green) for 2 cases (16.7%), deletion (purple) for one case (8.3%). Middle panel: Linear representation of the neurofibromin protein (2818 amino acids) with functional domains: CSRD (cysteine–serine-rich domain, aa 543–909), TBD (tubulin-binding domain, aa 1095–1187), GRD (GAP-related domain, aa 1198–1530), Sec14 homology domain (aa 1550–1728), PH (Pleckstrin Homology 1715–1816 aa) and CTD (C terminal domain, aa 2260–2818). Each mutation is positioned according to its amino acid residue. Symbols follow the color scheme from the upper panel: (●) nonsense, (■) frameshift, (▲) missense, (◆) deletion, (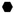) complex delins/novel variant (frameshift). The recurrent *NF1* c.910C>T (p.Arg304*) nonsense mutation was identified in two unrelated patients (P03 and P07). The novel frameshift variant *NF1* c.7504_7508delinsC (p.Ser2502Argfs*24) (P04) is located within the CTD. Lower panel: Clinical phenotype severity graded as mild (light green) for patients with only café-au-lait macules; moderate (yellow) for patients with additional cutaneous neurofibromas or FASI; and severe (red) for patients with plexiform neurofibromas, MPNST, or severe skeletal abnormalities. The novel variant (P04) is associated with the most severe clinical phenotype.

Following the molecular characterization of the cohort, the relationship between mutation types, protein domain localization, and clinical severity was assessed. The genotype–phenotype correlation in the Romanian NF1 cohort is presented in Figure 4. The figure integrates three levels of information: the distribution of mutation types (upper panel), the localization of each mutation on the neurofibromin protein domains (middle panel), and the corresponding clinical phenotype severity (lower panel) (Figure 4).

### 4.1. Mutational Diversity

The three novel frameshift mutations identified in this study (c.7504_7508delinsC, c.4967_4968insAGACT, and c.4625del) may contribute to a growing understanding of the mutational diversity in NF1. Their presence in unrelated patients with classic and severe phenotypes confirms that frameshift variants are a major driver of the disease. Furthermore, reporting these novel variants is essential for improving genotype–phenotype correlations and for refining diagnostic and prognostic algorithms in NF1. The discovery of a novel variant in a sporadic case underscores the importance of comprehensive genetic testing (even in the absence of a family history), and the value of reporting new mutations to enrich existing databases.

More recently, a distinct genotype–phenotype correlation has been established for splicing variants leading to in-frame skipping of *NF1* exon 24 (formerly known as exon 19a). Patients harboring these variants typically exhibit a milder phenotype, characterized by a lower frequency of neurofibromas and other severe NF1-specific features [29].

### 4.2. Recurrent Mutations

The recurrent nonsense variant *NF1* c.910C>T (p.Arg304*) was identified in two unrelated patients (P03 and P07) with discordant family histories (one sporadic and one familial). This variant is among the most frequently reported *NF1* mutations in the literature and is widely recognized as a mutational hotspot, given its location within a CpG dinucleotide in exon 9 where recurrent de novo events are well documented [30]. In our cohort, the recurrence of this known variant in two unrelated patients is noteworthy; however, further characterization through microsatellite or SNP-based haplotype analysis in a larger cohort of Romanian carriers would be required to determine whether this represents a population specific founder effect or a coincidental recurrence.

All 12 patients in this cohort presented with the classic NF1 phenotype, and no cases of atypical presentation such as neurofibromatosis type 1 with Noonan syndrome-like features (NFNS), were identified.

Patients carrying nonsense mutations (P03, P05, P07, and P12) and those with frameshift mutations (P01, P04, P06, P09, and P11) exhibited the classic NF1 phenotype without distinct clinical differences, suggesting that mutation type alone did not predict phenotypic severity within this cohort. Similarly, the missense variants (P02 and P08) and the single deletion (P10) were also associated with classic NF1, supporting the notion that a wide range of mutational events converge toward a common clinical spectrum. This observation stands in contrast to previous reports associating specific variant types or exons with atypical phenotypes, as no cases of NFNS were observed despite the presence of variants in exons 9 and 28, regions previously linked to more severe or atypical presentations [10]. Our findings are consistent with several recent studies that have reported similar discrepancies between predicted and observed phenotypes. For instance, Koczkowska et al. [12] found that not all individuals carrying high risk missense variants in exon 28 develop the full spectrum of severe manifestations, suggesting the influence of additional modifying factors [12]. Similarly, Pacot et al. [15] emphasized that the clinical expression of *NF1* mutations is highly variable and cannot be reliably predicted from the genotype alone [15]. Collectively, these findings suggest that additional factors, including modifier genes, somatic mosaicism, and epigenetic mechanisms, play a significant role in modulating the clinical expression of *NF1* mutations, a notion further supported by the marked variability in disease severity observed among patients carrying the same mutation (e.g., P03 and P07). This concept is reinforced by studies demonstrating that “second-hit” somatic events, epigenetic modifications, and genetic background significantly contribute to the wide phenotypic spectrum observed in NF1 [17]. These findings emphasize the complexity of genotype–phenotype correlations in NF1 and highlight the need for integrative approaches that consider both genetic and environmental contributors.

### 4.3. Genotype–Phenotype Correlations

All 12 patients presented with the classic NF1 phenotype, with variable expressivity across different organ systems. Patient P04, carrying the novel frameshift *NF1* c.7504_7508delinsC (p.Ser2502Argfs*24) variant, exhibited the most severe clinical phenotype, including multiple tumoral manifestations (cutaneous neurofibromas, plexiform neurofibroma, and MPNST), extensive musculoskeletal involvement (long bone dysplasia, vertebral dysplasia, and dystrophic scoliosis), neurological abnormalities, behavioral manifestations, and FASI on MRI. This suggests that the novel variant may be associated with a particularly severe disease course, although the small sample size precludes definitive conclusions. Patient P11, carrying a frameshift *NF1* c.4625del p.(Asn1542Thrfs*11) mutation, presented with a complex phenotype including freckling, Lisch nodules, cutaneous and plexiform neurofibromas, nondystrophic scoliosis, and FASI but without malignant transformation. Patients with nonsense mutations (P03, P05, P07, and P12) displayed heterogeneous phenotypes: P05 (*NF1* c.7285C>T, (p.Arg2429*) presented with freckling, Lisch nodules, hypertension, cardiovascular abnormalities, and FASI but no neurofibromas; P07 (*NF1* c.910C>T, p.(Arg304*) presented with behavioral manifestations, developmental delay, musculoskeletal abnormalities, and FASI; and patients P03 and P12 presented with milder phenotypes, primarily pigmentary manifestations and FASI. P08 (*NF1* c.4868A>T, p.(Asp1623Val) presented with optic pathway glioma, neurological abnormalities, cardiovascular abnormalities, and FASI. Patient P10, with a splicing deletion in exon 19, presented with optic pathway glioma and FASI, but no neurofibromas or other severe complications. FASI on MRI was highly prevalent (81.8%) across all mutation types, with no clear correlation with neurological symptoms or cognitive impairment (Table 1 and Table 2).

Patient P01 (D.A.C.), a 4-year-old male with a positive family history of NF1, presented with six or more café-au-lait macules, meeting the diagnostic criteria for NF1. No other major manifestations were observed. Genetic testing identified the frameshift *NF1* c.1329dup, p.(Gly444Trpfs*2) variant, also denoted p.(Gly444fs), in exon 12. This variant has been previously reported in European cohorts (PMIDs: 10712197 and 23913538) [27,28] and is listed in ClinVar (variation ID: 2002154) [26], but is absent from gnomAD [31].

This frameshift creates a premature translational stop signal p.(Gly444Trpfs*2), resulting in an absent or disrupted protein product. Loss-of-function (LOF) variants in *NF1* are well-established as pathogenic. Frameshift variants typically trigger nonsense-mediated mRNA decay, leading to complete loss of neurofibromin function, a mechanism strongly associated with classic NF1 phenotypes [27].

The absence of neurofibromas or skeletal abnormalities at age 4 is consistent with the age-dependent penetrance of these features. The phenotype aligns with previously described cases carrying LOF variants in exon 12 [28]. No genotype-specific correlation was noted for this variant, supporting the general observation that most truncating mutations lead to classic NF1 without distinct subphenotypes.

Patient P02 (G.T.), a 7-year- old male with a positive family history of NF1, exhibited café-au-lait macules, axillary freckling, behavioral manifestations, language delay, and FASI on brain MRI but no neurofibromas. The missense *NF1* c.3610C>G, p.(Arg1204Gly) variant in exon 27 affects the GAP-related domain (GRD), a critical region for Ras inactivation. Functional studies have demonstrated that this variant significantly reduces the GTPase activating activity of neurofibromin (PMIDs: 22807134 and 26635368) [32,33]. Missense variants in the GRD are generally associated with classic NF1, though some studies have reported a higher prevalence of cardiovascular abnormalities and cognitive deficits (PMIDs: 31595648 and 38362057) [14,34]. While P02 did not exhibit cardiovascular involvement, the presence of language delay and FASI aligns with the neurodevelopmental phenotype described for GRD missense mutations [10,12,15]. The absence of neurofibromas at age 7 is within the expected range of phenotypic variability.

The *NF1* c.3610C>G, p.(Arg1204Gly) variant is listed in The Human Gene Mutation Database (HGMD) [25], Leiden Open Variation Database (LOVD) [35], and ClinVar (Variation ID: 68337) [26] databases. In addition, another missense variant affecting the same codon, p.(Arg1204Trp), has been reported in NF1 patients (PMIDs: 22807134 and 10607834) [32,36].

Patient P03 (M.V.), a 2-year-old male with a negative family history, carries the recurrent *NF1* c.910C>T, p.(Arg304*) variant in exon 9 of the *NF1* gene. This pathogenic mutation is widely documented in international databases (ClinVar—variation ID: 187722; gnomAD; and LOVD) [26,31,35] and typically occurs as a de novo event due to a CpG mutational hotspot. Mechanistically, it triggers an LOF through exon 9 skipping and nonsense-mediated mRNA decay (PMID: 9463322) [37]. Clinically, P03 exhibits mild language delay and EEG abnormalities (isolated sharp waves), consistent with the increased frequency of cognitive deficits and cortical hyperexcitability reported in patients carrying this specific variant (PMIDs: 23668869, 30014477, 26509978, and 35589737) [38,39,40,41]. This phenotype reinforces the well-recognized association between the p.(Arg304*) mutation and significant neurodevelopmental manifestations.

Patient P04 (M.E.M.), an 11-year-old female with a negative family history of NF1, presented with an exceptionally severe phenotype including multiple plexiform neurofibromas, MPNST, extensive musculoskeletal abnormalities, and neurological deficits. The novel frameshift *NF1* c.7504_7508delinsC, p.(Ser2502Argfs*24) variant (in exon 54) has not been previously reported in the literature. This variant leads to premature protein truncation within the C-terminal domain (CTD) of neurofibromin, downstream of the GRD and TBD. Truncating variants in the C terminal region of neurofibromin have been associated with more severe phenotypes, including higher tumor burden and increased risk of malignant transformation (PMID: 35066574) [42]. However, we emphasize that this is a single severe case, and no causal association between this specific novel variant and the aggressive phenotype can be established based on one observation. The severity observed in P04 suggests a possible association that warrants further investigation in larger cohorts, but this interpretation remains strictly descriptive and hypothesis generating.

From a functional perspective, disruption of the CTD may impair microtubule binding and dysregulate alternative signaling pathways beyond Ras, potentially contributing to the aggressive clinical course [28]. Additionally, the presence of MPNST at a young age is a rare event in NF1, occurring in approximately 8–13% of patients over a lifetime [43], and is associated with truncating mutations in some series [44]. The severity observed in P04 suggests that this novel variant may confer a particularly high-risk phenotype, warranting close surveillance and early therapeutic intervention.

Patient P05 (P.S.), a 12-year-old female with a negative family history of NF1, carried the nonsense *NF1* c.7285C>T, p.(Arg2429*) variant in exon 50. This variant has been previously reported in several cohorts (PMIDs: 10712197,10678181, and 29872168) [27,45,46] and is listed in ClinVar (variation ID: 185789) [26]. It is absent from gnomAD v2 and generates a premature stop codon in exon 50, predicted to cause loss of normal protein function through nonsense-mediated mRNA decay (NMD)—an established disease mechanism in NF1.

The clinical presentation was dominated by congenital cardiovascular abnormalities (truncus arteriosus, ventricular septal defect, and right ventricular hypertrophy) and numerous cutaneous neurofibromas, with only a few café-au-lait spots—an unusual distribution for classic NF1. While cardiovascular defects occur in 2.3% to 12.6% of NF1 patients [47], the severity of P05’s heart disease is exceptional. Some genotype–phenotype studies have suggested an increased risk of cardiovascular abnormalities with missense variants in the GRD [15,48], but P05 carried a nonsense variant, indicating that additional modifying factors—either genetic (e.g., modifier genes) or environmental—likely contributed to her phenotype.

Patient P06 (D.A.), a 1-year-and-6-month-old male with a negative family history of NF1, carried the frameshift *NF1* c.4967_4968insAGACT, p.(Tyr1657Aspfs*22) variant in exon 36. This variant is absent from gnomAD has not been reported in the medical literature or on disease-related variation databases. It generates a premature stop codon, predicted to cause loss of normal protein function through nonsense-mediated mRNA decay (an established disease mechanism in NF1) and was classified as likely pathogenic (according to ACMG guidelines) [24]. The variant is located within the Sec14 homology domain (amino acids 1575–1745), a region involved in lipid binding and intracellular trafficking. Truncation of this domain may disrupt neurofibromin localization and protein–protein interactions, although the precise functional consequences remain unknown.

The clinical picture was complicated by concomitant fetal alcohol spectrum disorder (FASD), making it difficult to attribute developmental delay, dysmorphic features, and microcrania solely to NF1. While frameshift variants in this region have been associated with classic NF1 in several studies (PMIDs: 33919865 and 37217626) [11,49], no specific subphenotype has been delineated for variants in exon 36.

Large cohort studies have demonstrated that frameshift variants, are significantly associated with skeletal abnormalities (PMIDs: 33919865 and 10339848) [11,50]. The presence of café-au-lait macules supports the NF1 diagnosis, while the developmental delay may reflect contributions from both NF1 and FASD. This case highlights the challenge of phenotype interpretation in patients with dual diagnoses and underscores the importance of comprehensive genetic counseling.

Patient P07 (T.S.), a 2-year and 3- monthold male, carried the same recurrent nonsense variant as patient P03, *NF1* c.910C>T, p.(Arg304*). It is a well-recognized pathogenic germline mutation in the *NF1* gene, characterized by a premature translational stop signal that results in a truncated and likely nonfunctional neurofibromin protein. This variant is associated with the clinical diagnosis of NF1, which frequently includes cognitive and neurodevelopmental problems [40]. Unlike P03, patient P07 had a positive family history and exhibited a more severe neurodevelopmental phenotype, including autism spectrum disorder, intellectual disability (DQ 55%), expressive language disorder, and FASI on MRI. The clinical variability between P03 and P07, despite sharing the identical mutation, exemplifies the concept of variable expressivity in NF1, likely influenced by genetic modifiers, stochastic factors, or environmental exposures [51].

Patient P08 (C.O.), a 3-year-old female with a positive family history of NF1, carried the missense *NF1* c.4868A>T, p.(Asp1623Val) variant in exon 33. The clinical presentation was notable for an optic pathway glioma (OPG) involving the left optic nerve and chiasm, along with FASI but no neurofibromas. OPGs occur in approximately 15–20% of children with NF1 [52,53] and result from loss of the neurofibromin protein, leading to overactivation of the RAS signaling pathway [53]. Xu et al. [54] demonstrated an association between *NF1* mutations and an increased risk of OPGs, with most associated variants being missense mutations, and identified the CSRD as a high-risk “hotspot” region. In the same study, two patients with OPGs carried large deletions involving exons 30–34 and exons 31–35, respectively [54]. The *NF1* c.4868A>T, p.(Asp1623Val) variant is located in the GRD region of the *NF1* gene. It is not reported in gnomAD [31] but is listed in ClinVar (variation ID: 2765159) [26]. This variant has not been reported in the medical literature in patients with NF1-related disease. Assessment of experimental evidence suggests that this variant results in abnormal protein function (PMID: 34948100) [55].

Other missense variants in the same codon, p.(Asp1623Tyr), p.(Asp1623Gly), p.(Asp1623His), p.(Asp1623Asn), and p.(Asp1623Ala), have been reported in individuals with NF1-related phenotypes, suggesting that this residue is clinically significant and that variants disrupting it are likely disease-causing (PMIDs: 23913538, 23656349, 34694046, and 26740943) [28,56,57,58]. The absence of neurofibromas at age 3 is consistent with the age-dependent onset of these lesions. The positive family history confirms germline transmission.

Patient P09 (S.A.), a 4-month-old female, with a negative family history of NF1, carried the frameshift *NF1* c.499_502del, p.(Cys167Glnfs*10) variant in exon 5, resulting in a premature codon stop and loss of protein function. This is a well-recognized pathogenic variant, listed in several databases including ClinVar (variation ID: 185021) [26], HGMD [25], and LOVD [35]. The mutation has been reported in several studies in the literature (PMIDs: 10543400, 12807981, and 17311297) [59,60,61], occurring as a sporadic (de novo) event in most cases.

Patient P09 presented with congenital pseudarthrosis of the tibia (CPT), a severe skeletal complication occurring in 3–5% of NF1 patients [62,63]. CPT has been associated with truncating variants in the first half of the gene in several studies [64,65,66].

Recent studies have confirmed that the location of mutations in the 5′ region of the *NF1* gene (specifically in the first tertile, corresponding to exons 1–10) represents a critical prognostic indicator, being strongly correlated with a severe phenotype that includes a high incidence of CPT, early-onset OPG, and an increased overall tumor risk [67].

The early-onset of CPT in P09 aligns with these observations and suggests that the location of the mutation may influence the risk of skeletal manifestations. The presence of FASI on brain MRI, even at this young age, is a common finding in NF1 and does not predict neurological outcome.

Patient P10 (R.M.G.), an 8-year-old female with a negative family history of NF1, carried a deletion of exon 19 (*NF1* c.(2809 −?_2905+?1)del). The clinical phenotype was characterized by optic nerve neurofibroma, FASI, chronic kidney disease (CKD) with secondary hypertension, and dorsal kyphosis, with no cutaneous neurofibromas. Intragenic deletions in *NF1* are generally associated with classic NF1, though some studies have reported a higher prevalence of cardiovascular and skeletal abnormalities (PMIDs: 28213670 and 34199217) [68,69]. Although some studies have suggested that NF1 mutations located in the 5′ tertile (exons 1–21) or affecting the cysteine/serine-rich domain (CSRD) may be associated with an elevated risk of optic pathway gliomas [53,67], this genotype–phenotype correlation remains unconfirmed in larger independent cohorts, and current evidence does not support its use in clinical risk stratification [70,71]. Indeed, the present case, with a deletion of exon 19 (a region encompassed by the 5′ tertile) but presenting with an optic nerve neurofibroma rather than a glioma, exemplifies this phenotypic heterogeneity.

The absence of cutaneous neurofibromas at age 8 years is within the expected clinical course, as these tumors typically appear during adolescence and often continue to grow in size and multiply into adulthood [2]. The presence of optic nerve neurofibroma (as opposed to OPG) is less common but well documented in NF1, particularly in their plexiform form with potential to cause secondary glaucoma [72,73]. This case illustrates the phenotypic variability associated with intragenic deletions.

Patient P11 (U.M.I.), a 13-year-old female with a negative family history of NF1, carried the frameshift *NF1* c.4625del, p.(Asn1542Thrfs*11) variant in exon 34. She presented with a progressive left brachial plexus plexiform neurofibroma (partially resected), multiple spinal neurofibromas, scoliosis, and postoperative left Horner syndrome. Frameshift variants, including those located in the central region of the *NF1* gene (exons 30–35), have been associated with loss-of-function (LOF) and are frequently observed in patients with plexiform neurofibromas, although no specific exon-level hotspot has been definitively established [54,74,75].

The *NF1* c.4625del, p.(Asn1542Thrfs*11) variant is a rare, likely pathogenic mutation that is not present in major population databases (ClinVar, LOVD, and gnomAD), making it highly unlikely to be a common benign variant in the general population.

However, as a loss-of-function (LOF) mutation leading to premature protein termination—a class in which over 99% of *NF1* variants are pathogenic or likely pathogenic—this variant is highly likely to be disease-causing [2,76]. This frameshift variant p.(Asn1542Thrfs*11) results in a truncated neurofibromin protein lacking the functional GRD, which is essential for Ras-GAP activity. This loss may affect neurofibromin’s interactions with other signaling pathways, potentially contributing to the aggressive tumor phenotype [77]. Given the significant tumor burden and disease progression, selumetinib therapy was initiated in this patient.

Patient P12 (M.S.), a 7-month-old female, with positive family history of NF1, carried the nonsense *NF1* c.236T>A, p.(Leu79*) variant in exon 2. She presented with multiple café-au-lait macules and FASI, with early involvement of the left optic tract on MRI but no clinical signs of OPG.

The *NF1* c.236T>A (p.Leu79*) variant is a nonsense mutation that introduces a premature stop codon at amino acid position 79. This leads to a truncated neurofibromin protein, which is typically degraded through nonsense-mediated mRNA decay (NMD), resulting in a complete loss-of-function. Although this specific c.236T>A substitution is not currently listed in international databases, such as ClinVar or gnomAD, the same premature stop codon is caused by the *NF1* c.236T>G (p.Leu79*) variant. This alternative variant has been identified in two patients with NF1 (PMID: 31766501) [70] and is listed in ClinVar (variation ID: 655362) [26]. As a nonsense mutation, this variant is predictably pathogenic. This aligns with the well-established paradigm that premature stop codons in NF1 almost invariably result in loss-of-function and clinical disease.

Some studies have suggested that genotype is the main determinant of OPG development, with an increased risk observed in patients harboring *NF1* mutations within the 5′ tertile or the CSRD [53,54,67]. However, contradictory results have been reported by several independent studies. Hutter et al. [71] analyzed a cohort of 77 patients and did not confirm clustering of mutations in the 5′ region among those with OPGs [71]. Similarly, Melloni et al. [70] combined their own cohort of 309 patients with a previously described cohort of 381 patients, for a total of 690 patients. Upon statistical re-analysis, they found no correlation between mutation location (tertile or domain) and OPG risk, thereby refuting the initial conclusions [70]. Due to conflicting evidence from large-scale studies, current clinical guidelines do not support OPG risk stratification solely based on *NF1* mutation location [71]. Our findings, as illustrated in Figure 4, support this lack of clustering, given that our patients with optic pathway involvement (P08, P10, and P12) harbor variants distributed across different regions of the neurofibromin protein (the GRD, exon 19, and the 5′ tertile, respectively).

The early detection of optic tract involvement in patient P12 highlights the importance of systematic neuroimaging in young NF1 patients, even in the absence of clinical symptoms. The positive family history (affected mother) confirms autosomal dominant inheritance. Furthermore, the absence of neurofibromas at 7 months is expected, as these lesions typically appear later in childhood.

Within the molecularly confirmed subgroup (*n* = 12), brain MRI was available for 11 patients. Focal abnormal signal intensities (FASIs) were present in 9 of these 11 patients (81.8%), consistent with published data reporting FASI in 60–90% of children with NF1 [78]. Notably, in the larger clinically diagnosed cohort (*n* = 54), FASI were reported in 27 patients (50.0%), reflecting the broader clinical spectrum. In the molecular subgroup, no clear correlation was observed between FASI burden and specific neurocognitive deficits.

For instance, patient P02 presented with FASI and language delay, while patient P06 had FASI but no cognitive impairment (his developmental delay was primarily attributed to fetal alcohol spectrum disorder). Patient P07 exhibited both FASI and severe neurodevelopmental manifestations (autism spectrum disorder and intellectual disability), whereas patient P03 had FASI and only mild language delay. While FASI were already identifiable in the youngest patients (e.g., P09, aged 4 months, and P12, aged 7 months), supporting their early emergence, age-related trends could not be reliably assessed due to the cross-sectional nature of the data. Larger prospective studies are needed to clarify whether FASI burden or location predicts long-term neurocognitive outcomes.

### 4.4. Study Limitations

The present study has several limitations that should be acknowledged.

*Cohort size and generalizability.* The cohort size is relatively small (*n* = 12 for the molecularly confirmed subgroup), which limits statistical power for robust genotype–phenotype correlations and precludes generalization to the broader Romanian NF1 population. Consequently, our findings should be regarded as exploratory and hypothesis-generating rather than definitive. They serve as a foundational step for future multicenter initiatives, not as a comprehensive characterization of NF1 in Romania.

*Selection bias and representativeness.* Importantly, the 12 molecularly confirmed patients represent a subgroup of a larger clinically diagnosed cohort (*n* = 54). Selection was driven by the availability of complete molecular testing (NGS + MLPA), which in turn was influenced by financial constraints (genetic testing for NF1 is not covered by the Romanian national health system) and by the retrospective nature of this study (many patients were diagnosed before the 2021 NIH criteria mandated molecular testing). Consequently, the molecularly confirmed subgroup may not be fully representative of the entire Romanian NF1 population. Patients from lower socioeconomic backgrounds, who could not afford NGS, are likely underrepresented. This selection bias should be carefully considered when interpreting the genotype–phenotype correlations reported here. Moreover, this limitation reflects real-world challenges in resource-limited settings and highlights the urgent need for national funding of genetic testing for rare diseases, including NF1.

*Retrospective design and heterogeneity of clinical data.* The long-term retrospective design (2016–2026) means that many patients were evaluated when diagnosis was based solely on the 1987 NIH clinical criteria, before genetic confirmation was integrated into the guidelines in 2021. As a result, earlier patients often lacked the indication for molecular testing. Furthermore, clinical data were collected from medical records without a fully standardized set of investigations; neuropsychological assessments, uniform imaging protocols, and longitudinal follow-up data were not available for every patient. This heterogeneity may have introduced bias in phenotype severity grading.

*Lack of functional and RNA studies*. Functional studies to assess the impact of the novel frameshift variant identified in patient P04 (c.7504_7508delinsC, p.Ser2502Argfs*24) were not performed. Similarly, the absence of RNA analysis for splicing variants may have led to an underestimation of splicing defects, which account for a significant proportion of NF1 mutations [28,36,74].

*Detection limitations for mosaicism and deep intronic variants*. Our methodology (targeted NGS panel and MLPA) was not designed to detect low-level mosaicism or deep intronic variants not affecting splice sites. While these are relatively rare in classic, non-mosaic NF1, their presence cannot be formally excluded. Future studies incorporating whole-genome sequencing (WGS), RNA analysis, and testing of affected tissues (when available) will be required to address this gap.

*Absence of a control group*. We did not include an independent control group (e.g., patients with suspected NF1 but negative genetic testing), which would have allowed calculation of the sensitivity and specificity of clinical criteria in our population. Incorporating a symptomatic, genotype-negative control group should be considered in future larger-scale initiatives.

*Exclusion of alternative RASopathies*. Despite the clinical overlap of NF1 with other RASopathies (such as Legius syndrome, Noonan syndrome, and PTEN hamartoma syndrome), all patients in our cohort met the revised 2021 NIH diagnostic criteria for NF1, and alternative diagnoses were excluded clinically before genetic testing.

### 4.5. Significance as the First Romanian NF1 Cohort Study

To our knowledge, this is the first study to provide a combined molecular and clinical characterization of a Romanian cohort of patients with NF1. In Romania, published research on neurofibromatosis type 1 is scarce and dominated by case reports and small clinical studies. The 2007 study by Buteică et al. [79], which reported six cases (three sporadic), is one of the few addressing the genetic aspect [79]. However, consistent with the resources available at the time, it only describes the inheritance pattern (de novo vs. inherited) without identifying or reporting the specific genetic variants. As such, it represents a foundational step toward understanding the mutational spectrum and clinical heterogeneity of NF1 in Romania. The identification of three novel variants and the recurrence of *NF1* c.910C>T (p.Arg304*) variant in two unrelated patients highlights the need for larger, multicenter studies to further characterize population-specific mutational patterns in the Romanian cohort.

Such studies would enable comparisons with other European countries and ethnically diverse populations, potentially revealing population specific mutational hotspots and genotype–phenotype patterns [3,28,50,67].

Beyond the molecular findings, this study provides the first systematic clinical characterization of a large cohort of Romanian patients with NF1 (*n* = 54). To the best of our knowledge, no previous report has documented the frequency of major clinical manifestations within a Romanian NF1 population.

The observed frequencies (100% for café-au-lait macules, 64.8% for Lisch nodules, 61.1% for axillary freckling, 42.6% for cutaneous neurofibromas, 50.0% for FASI on MRI, 20.4% for nondystrophic scoliosis, and 27.8% for plexiform neurofibromas) are broadly consistent with published data from other European cohorts [80,81,82,83].

This clinical baseline is highly valuable for local healthcare planning, for educational and training purposes, and as a reference point for future comparative studies in Central and Eastern Europe.

### 4.6. Importance of Early Molecular Diagnosis and Genetic Counseling

A key finding of this study is the high proportion of sporadic cases (7 out of 12, 58.3%), underscoring the importance of molecular genetic testing for establishing an accurate diagnosis, particularly in young children who may not yet fulfill all clinical diagnostic criteria. In our cohort, patients diagnosed at very young ages—such as P09 (4 months), P12 (7 months), P06 (1 year and 6 months), and P03 (2 years)—benefited from early genetic confirmation, which facilitated timely clinical surveillance and family counseling. In these cases, testing was indicated either by a positive family history (P12) or by the presence of multiple café-au-lait macules (P03, P06, and P09), reinforcing the utility of genetic testing as a first line diagnostic tool in the pediatric population.

Genetic counseling was offered to all families enrolled in this study, with particular emphasis on explaining the autosomal dominant inheritance pattern, the 50% recurrence risk for future offspring, and the variable expressivity that characterizes NF1. For sporadic cases, parents were counseled regarding the de novo nature of the mutation and the low recurrence risk in subsequent pregnancies, while for familial cases, cascade testing of at-risk relatives was recommended. The provision of accurate genetic counseling is essential for informed reproductive decision making and for ensuring appropriate long-term medical follow up.

The discrepancy between the number of clinically diagnosed patients (*n* = 54) and those with complete molecular confirmation (*n* = 12) highlights a critical gap in access to genetic testing for NF1 in Romania. Financial constraints were the primary barrier, as NGS is not reimbursed by the national health system, and even MLPA testing was discontinued due to lack of funding. This situation is likely not unique to Romania but reflects broader challenges in Central and Eastern European countries. Addressing these disparities should be a priority for healthcare policymakers, as molecular confirmation is essential not only for accurate diagnosis and genetic counseling but also for determining eligibility for targeted therapies (e.g., selumetinib for plexiform neurofibromas).

### 4.7. Therapeutic Perspectives: Targeted Therapy for Plexiform Neurofibromas

The identification of patients with progressive plexiform neurofibromas in our cohort (P04 and P11) highlights the importance of recognizing candidates for targeted therapy. Selumetinib, a selective MEK1/2 inhibitor, has been approved for the treatment of symptomatic, inoperable plexiform neurofibromas in children with NF1 based on the phase II SPRINT trial, which demonstrated durable tumor volume reduction and clinical improvement in pain and quality of life [16,84]. In our study, patient P04 was undergoing selumetinib therapy for a large lumbar plexiform neurofibroma with significant neurological compromise, while patient P11 was being considered for treatment due to disease progression. These cases illustrate the transformative impact of molecularly targeted therapies in NF1 and underscore the need for early referral to specialized multidisciplinary centers.

In conclusion, this study operates on two complementary levels. First, we provide a comprehensive clinical baseline of NF1 in a Romanian cohort of 54 patients, documenting the frequency of major diagnostic criteria and complications. Second, within a molecularly confirmed subgroup of 12 patients, we report three novel *NF1* variants and establish preliminary genotype–phenotype correlations. While the small size of the molecular subgroup limits statistical power, the clinical description of the larger cohort adds valuable context and illustrates the real-world challenges of accessing genetic testing.

### 4.8. Future Directions

The present study lays the groundwork for several future research directions. First, expansion to a multicenter national registry would enable the collection of clinical and molecular data from a larger cohort of Romanian NF1 patients, allowing for robust genotype–phenotype correlation analyses and the identification of population-specific mutational patterns. Future studies should be framed within recent neurogenetic precision medicine approaches, where genotype is integrated with standardized phenotype, longitudinal follow up, modifier assessment, imaging/functional data, and explicit uncertainty, rather than used as a deterministic predictor of individual outcome [85].

Second, future studies should adopt a prospective, multicenter design with standardized, blinded phenotyping protocols, predefined inclusion and exclusion criteria, and independent validation of novel variants in control populations. Third, whenever possible, functional studies (e.g., RNA sequencing, minigene assays, and Western blot for protein expression) and multi-tissue testing for mosaicism should be incorporated to improve the characterization of splice variants and the novel frameshift variants identified here. Fourth, longitudinal follow-up of the cohort will provide valuable data on the natural history of the disease and the long-term outcomes of targeted therapies. Fifth, a separate forthcoming study will provide a detailed analysis of the clinical, epidemiological, and treatment-related outcomes of the entire 54-patient clinically diagnosed cohort, including longitudinal follow-up and a comprehensive evaluation of the 42 patients who lacked complete molecular confirmation.

Finally, comparative studies with other European populations would help delineate the unique features of NF1 in Romania and contribute to the global understanding of this complex disorder.

## 5. Conclusions

This study provides the first dual-level characterization of NF1 in a Romanian cohort: a clinical description of 54 patients meeting diagnostic criteria, followed by preliminary molecular confirmation and descriptive, exploratory genotype–phenotype observations in a subgroup of 12 patients. This establishes a foundational framework for future national research in a population previously underrepresented in the international literature.

Within the molecularly confirmed subgroup, frameshift and nonsense mutations accounted for 75% of the variants, including three novel frameshift mutations. The recurrent nonsense variant c.910C>T (p.Arg304*) observed in two unrelated patients is consistent with a known mutational hotspot; however, haplotype analysis in a larger cohort is required before any population specific interpretation can be made. The high proportion of sporadic cases (58.3%) underscores the vital role of early molecular testing, particularly in infants and young children presenting with isolated café-au-lait macules. Early genetic confirmation facilitated timely multidisciplinary surveillance, accurate prognostic assessment, and comprehensive family counseling.

The identification of severe phenotypes, including progressive plexiform neurofibromas and a malignant peripheral nerve sheath tumor, highlights the urgency of early referral to specialized centers and illustrates the emerging role of targeted therapies, such as the MEK inhibitor selumetinib.

Despite the acknowledged limitations (including small cohort size, retrospective design, and lack of functional studies—which preclude definitive genotype–phenotype correlations), this first regional report from Romania offers valuable data for cross-population comparisons and represents an essential starting point for improving diagnostic accuracy and genetic counseling for NF1 patients in this region. Future prospective studies incorporating transcriptome sequencing, more sensitive mosaicism assays, and standardized clinical evaluations are needed to fully capture the molecular and clinical heterogeneity of NF1.

## Figures and Tables

**Table 1:** Pathogenic and likely pathogenic NF1 variants identified in the molecularly confirmed subgroup (*n* = 12).

Patient ID	Mutation/Variant	Transcript	Exon (E)	Protein	Genotype	Reported in the Literature/Databases	Effect/Pathogenicity (ACMG)	Family History of NF1
P01 (D.A.C)	*NF1* c.1329dup	NM_000267.3	E12	(p.Gly444Trpfs*2)	Hz	Known	Frameshift/Pathogenic	Positive
P02 (G.T)	*NF1* c.3610C>G	NM_000267.3	E27	p.(Arg1204Gly)	Hz	Known	Missense/Pathogenic	Positive
P03 (M.V.)	*NF1* c.910C>T	NM_000267.3	E9	p.(Arg304*)	Hz	Known	Nonsense/ Pathogenic	Negative
P04 (M.E.M)	*NF1* c.7504_7508delinsC	NM_000267.3	E54	p.(Ser2502Argfs*24)	Hz	New	Frameshift/Pathogenic	Negative
P05 (P.S.)	*NF1* c.7285C>T	NM_000267.3	E50	p.(Arg2429*)	Hz	Known	Nonsense/ Pathogenic	Negative
P06 (D.A.)	*NF1* c.4967_4968insAGACT	NM_000267.3	E36	p.(Tyr1657Aspfs*22)	Hz	New	Frameshift/Likely Pathogenic	Negative
P07 (T.S.)	*NF1* c.910C>T	NM_000267.3	E9	p.(Arg304*)	Hz	Known	Nonsense/ Pathogenic	Positive
P08 (C.O.)	*NF1* c.4868A>T	NM_000267.3	E33	p.(Asp1623Val)	Hz	Known	Missense/Likely pathogenic	Positive
P09 (S.A.)	*NF1* c.499_502del	NM_001042492.3	E5	p.(Cys167Glnfs*10)	Hz	Known	Frameshift/ Pathogenic	Negative
P10 (R.M.G)	*NF1* c.(2809 −?_2905+?1)del	NM_000267.3	E19		Hz	Known	Deletion/Pathogenic	Negative
P11 (U.M.I.)	*NF1* c.4625del	NM_000267.3	E34	p.(Asn1542Thrfs*11)	Hz	New	Frameshift/Likely Pathogenic	Negative
P12 (M.S.)	*NF1* c.236T>A	NM_000267.3	E2	p.(Leu79*)	Hz	Known	Nonsense/Pathogenic	Positive

Hz: heterozygous genotype; NF1: neurofibromatosis type 1; E: exon; ACMG: American College of Medical Genetics and Genomics.

**Table 2:** Clinical manifestations by mutation type in the molecularly confirmed NF1 subgroup (*n* = 12).

Clinical Feature	Frameshift (*n* = 5)	Nonsense (*n* = 4)	Missense (*n* = 2)	Deletion (*n* = 1)	Total (*n* = 12)
Mean age (years)	5.4	4.2	5.0	8.0	5.8
Sex (M/F)	2 M/3 F	2 M/2 F	1 M/1 F	0 M/1 F	5 M/7 F
Café-au-lait macules (≥6)	5 (100%)	4 (100%)	2 (100%)	1 (100%)	12 (100%)
Axillary/inguinal freckling	3 (60%)	1 (25%)	2 (100%)	1 (100%)	7 (58.3%)
Lisch nodules	2 (40%)	1 (25%)	0 (0%)	1 (100%)	4 (33.3%)
Optic pathway glioma (OPG)	0 (0%)	0 (0%)	1 (50%)	1 (100%)	2 (16.7%)
Cutaneous neurofibromas	2 (40%)	1 (25%)	0 (0%)	0 (0%)	3 (25%)
Plexiform neurofibromas	2 (40%)	0 (0%)	0 (0%)	0 (0%)	2 (16.7%)
MPNST	1 (20%)	0 (0%)	0 (0%)	0 (0%)	1 (8.3%)
Musculoskeletal abnormalities	3 (60%)	2 (50%)	0 (0%)	1 (100%)	6 (50%)
FASI on brain MRI (prevalence)	2/4 (50%) *	4/4 (100%)	2/2 (100%)	1/1 (100%)	9/11 (81.8%) *
Neurological/behavioral abnormalities	4 (80%)	2 (50%)	1 (50%)	1 (100%)	8 (66.66%)
Developmental delay/Intellectual disability	1 (20%)	1 (25%)	0 (0%)	0 (0%)	2 (16.7%)
Hypertension	0 (0%)	1 (25%)	0 (0%)	0 (0%)	1 (8.33%)
Other cardiovascular abnormalities	0 (0%)	1 (25%)	1 (50%)	0 (0%)	2 (16.7%)
Positive family history	1 (20%)	2 (50%)	2 (100%)	0 (0%)	5 (41.7%)
Sporadic (de novo) cases	4 (80%)	2 (50%)	0(0%)	1 (100%)	7 (58.3%)

* FASI prevalence: Brain MRI was available for 11 out of 12 patients (patient P01 had no available imaging). Among these, only P06 and P11 did not present FASI. Thus, 9 out of 11 patients (81.8%) with available MRI showed FASI; M: male; F: female; FASI: focal abnormal signal intensities; MPNST: malignant peripheral nerve sheath tumor; NF1: neurofibromatosis type 1; OPG: optic pathway glioma.

## Data Availability

The original contributions presented in this study are included in the article. Further inquiries can be directed to the corresponding author.

## Supplementary Materials

The following supporting information can be downloaded at https://www.mdpi.com/article/10.3390/genes17070843/s1, Supplementary Table S1. ACMG/AMP criteria applied for variant classification.

## Abbreviations

The following abbreviations are used in this manuscript:

NF1	Neurofibromatosis type 1
MPNST	Malignant peripheral nerve sheath tumor
CSRD	Cysteine–serine-rich domain
GRD	GAP-related domain
TBD	Tubulin-binding domain
NFNS	Neurofibromatosis type 1 with Noonan Syndrome-like features
OPG	Optic pathway glioma
CPT	Congenital pseudarthrosis of the tibia
