# Supplementary material for: Toward More Accurate Diagnosis in Neurofibromatosis Type 1: A Dual-Level Analysis of Clinical and Molecular Data with Exploratory Genotype–Phenotype Correlations in a Romanian Cohort
Source: Genes (Basel). 2026 Jul 22;17(7):843. doi: 10.3390/genes17070843 (PMC13411349; doi:10.3390/genes17070843)
Supplement: Supplementary file 1 [file genes-17-00843-s001.zip › genes-4413578-supplementary.pdf]

Supplementary Table S1. ACMG/AMP Criteria Applied for Variant Classification

Patient ID	Variant	Protein	Novel / Reported	ACMG/AMP Criteria Applied	Classification
P01	*NF1* c.1329dup	p.(Gly444Trpfs*2)	Reported	PVS1, PM2	Pathogenic
P02	*NF1* c.3610C>G	p.(Arg1204Gly)	Reported	PS1, PM2, PP3	Pathogenic
P03	*NF1* c.910C>T	p.(Arg304*)	Reported	PVS1, PS1, PM2	Pathogenic
P04	*NF1* c.7504_7508delinsC	p.(Ser2502Argfs*24)	Novel	PVS1, PM2, PM4	Pathogenic
P05	*NF1* c.7285C>T	p.(Arg2429*)	Reported	PVS1, PS1, PM2	Pathogenic
P06	*NF1* c.4967_4968insAGACT	p.(Tyr1657Aspfs*22)	Novel	PVS1, PM2, PM4	Likely Pathogenic
P07	*NF1* c.910C>T	p.(Arg304*)	Reported	PVS1, PS1, PM2	Pathogenic
P08	*NF1* c.4868A>T	p.(Asp1623Val)	Reported	PS1, PM2, PP3	Likely Pathogenic
P09	*NF1* c.499_502del	p.(Cys167Glnfs*10)	Reported	PVS1, PM2, PM4	Pathogenic
P10	*NF1* c.(2809-?_2905+?1)del	(exon 19 deletion)	Reported	PVS1, PM2	Pathogenic
P11	*NF1* c.4625del	p.(Asn1542Thrfs*11)	Novel	PVS1, PM2, PM4	Likely Pathogenic
P12	*NF1* c.236T>A	p.(Leu79*)	Novel nucleotide (same protein change reported)	PVS1, PM2	Pathogenic

PVS1: Null variant (frameshift, nonsense, deletion) in a gene where loss-of-function is a known mechanism of disease; PS1: Same amino acid change as a previously established pathogenic variant; PM2: Absent from population databases (gnomAD); PM4: Protein length change due to in-frame deletion/insertion or frameshift; PP3: Multiple in silico predictors support a deleterious effect
